# Overview of Approaches to Increase the Electrochemical Activity of Conventional Perovskite Air Electrodes

**DOI:** 10.3390/ma16144967

**Published:** 2023-07-12

**Authors:** Elena Filonova, Elena Pikalova

**Affiliations:** 1 Department of Physical and Inorganic Chemistry, Institute of Natural Sciences and Mathematics, Ural Federal University, Yekaterinburg 620002, Russia; 2 Laboratory of Kinetics, Institute of High Temperature Electrochemistry, Ural Branch of the Russian Academy of Sciences, Yekaterinburg 620137, Russia; e.pikalova@list.ru; 3 Department of Environmental Economics, Graduate School of Economics and Management, Ural Federal University, Yekaterinburg 620002, Russia

**Keywords:** SOFC, cathode, air electrode, LSM, LSCF, composite electrode, conductivity, infiltration, collector layer

## Abstract

The progressive research trends in the development of low-cost, commercially competitive solid oxide fuel cells with reduced operating temperatures are closely linked to the search for new functional materials as well as technologies to improve the properties of established materials traditionally used in high-temperature devices. Significant efforts are being made to improve air electrodes, which significantly contribute to the degradation of cell performance due to low oxygen reduction reaction kinetics at reduced temperatures. The present review summarizes the basic information on the methods to improve the electrochemical performance of conventional air electrodes with perovskite structure, such as lanthanum strontium manganite (LSM) and lanthanum strontium cobaltite ferrite (LSCF), to make them suitable for application in second generation electrochemical cells operating at medium and low temperatures. In addition, the information presented in this review may serve as a background for further implementation of developed electrode modification technologies involving novel, recently investigated electrode materials.

## 1. Introduction

The development of renewable energy resources, including hybrid power systems [1,2,3,4], is one of the ways for sustainable progress towards decarbonization [5,6,7]. Hybrid energy systems combining solid oxide fuel cells (SOFCs) with heat generators, energy storage devices, internal combustion engines, and solar cells have been intensively designed and manufactured today [4,8,9,10,11]. The progressive trend in the development of SOFCs is to lower the operating temperature, which brings undoubted advantages on the way to the commercialization of these power sources, such as the use of cheaper materials, faster start-up, and increased lifetime due to the reduction of degradation processes. However, challenges arise at low operating temperatures related to the slowing down of electrode reaction kinetics and the increasing ohmic resistance of the electrolyte membrane, resulting in a reduction in the SOFC performance [12,13]. To maintain the electrochemical performance of SOFCs operating at low (LT) and intermediate (IT) temperatures at a satisfactory level, the material optimization has been considered for all construction parts of the SOFC, such as cathodes [14,15,16,17,18,19], anodes [14,19,20,21] and electrolytes [19,22,23,24]. The cathode has been shown to be the major contributor to the electrochemical degradation of the cell [25]. The characterization and performance of the wide range of cathodes can be found in recent reviews [13,26,27,28,29].

An analysis of the literature data on the electrical, thermal, mechanical, and electrochemical properties of the conventional perovskite-type cathode materials shows that lanthanum strontium manganite (La,Sr)MnO_3_ (LSM) fulfils all the requirements for its use in high-temperature SOFCs [26,27]. However, as the temperature decreases, the use of LSM materials, which are predominantly electronic conductors with a low level of ionic conductivity, becomes unsatisfactory due to their low electrochemical activity for the oxygen reduction reaction (ORR) [30,31]. On the other hand, cobalt-based perovskite materials, including lanthanum strontium cobaltite ferrite (La,Sr)(Co,Fe)O_3 − δ_ (LSCF), are characterized by superior catalytic activity [32] due to high values of both electronic and ionic conductivity [33]. However, these materials exhibit increased thermal and chemical expansion, which is detrimental to the long-term operation of SOFCs [33,34]. The poor long-term durability of high-temperature electrochemical cells is often caused by the performance degradation phenomenon of the air electrode [35,36,37]. It has been found that the electrochemical performance degradation of the LSM- and LSCF-based air electrodes may include microstructural coarsening [33,38,39], the electrolyte/cathode interface reactions [33,40,41,42], sulfur [43,44,45] and chromium poisoning [44,46,47,48,49], carbon deposition [50,51], and Sr surface segregation [33,35,36,52].

**Figure 1:**
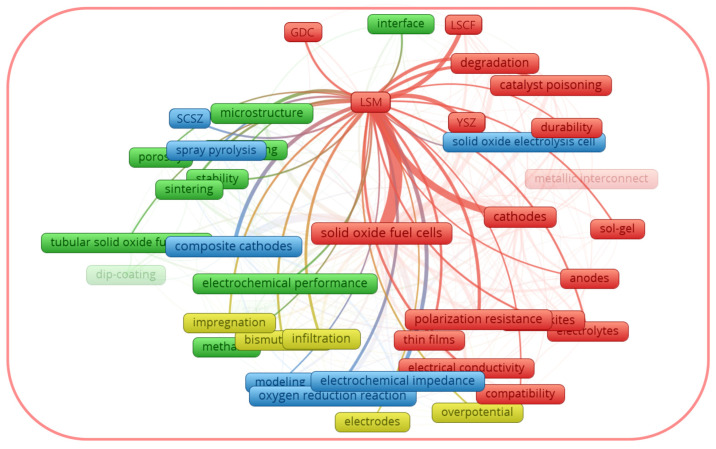
Thematic map of co-occurring author keywords in the Scopus dataset for (La,Sr)MnO_3_ (LSM).

**Figure 2:**
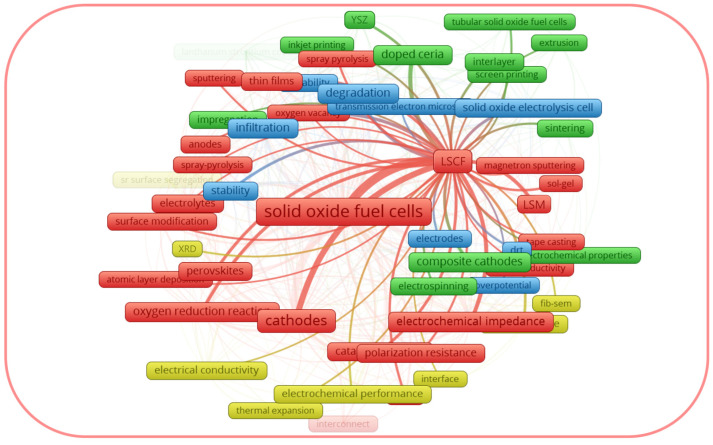
Thematic map of co-occurring author keywords in the Scopus dataset for (La,Sr)(Co,Fe)O_3 − δ_ (LSCF).

The perceived drawbacks of lanthanum strontium-based cathodes have driven research trends towards alternative solutions to improve their electrochemical performance and durability. A search of the Scopus database using the combination of the keywords “LSM”, “SOFC*” and “LSCF”, “SOFC*” (with further application of the limits of “cathode*”, “cathode materials”, “composite cathode*”, “cathode polarization”, “cathode performance”, “electrode*”, “oxygen electrode”, “electrochemical electrodes”) yielded 1141 and 1208 documents respectively for the period of 1996 for LSM and 1998 for LSCF to June of 2023. Figure 1 and Figure 2, generated with the software package *VOSviewer* version 1.6.19 [53], considering a minimum number of occurrences equal to five author keywords, visualize the maps with topic clusters related to LSM and LSCF as cathode materials for SOFCs, respectively.

From the graphical data shown in Figure 1, the author keywords of the documents considered as LSM can be divided into four clusters: the red cluster, focused on the degradation of the cells; the blue cluster, focused on the composite electrodes; the green cluster, focused on the cathode microstructure; and the yellow cluster, focused on the electrode modification using infiltration. Thus, the red cluster generalizes the drawbacks of the LSM cathode, while the other three clusters reflect the strategies to overcome them. From the graphical data shown in Figure 2, the author keywords of the documents considered as LSCF can be divided into four clusters, some of which are different from those for LSM. In the case of LSCF, the red, blue, and the green clusters generalize the LSCF performance under the operating conditions of fuel cells, electrolysis cells, and microtubular SOFCs, respectively. The yellow cluster generalizes the interfacial characterization. It is interesting to note that each cluster for LSCF includes the topic of drawbacks and their possible solutions. Thus, the above-mentioned trends justify that the improvement of the activity of LSM and LSCF electrodes remains prominent, and it is important to summarize the thematic studies, provided that the performance of LSM and LSCF has been improved.

**Figure 3:**
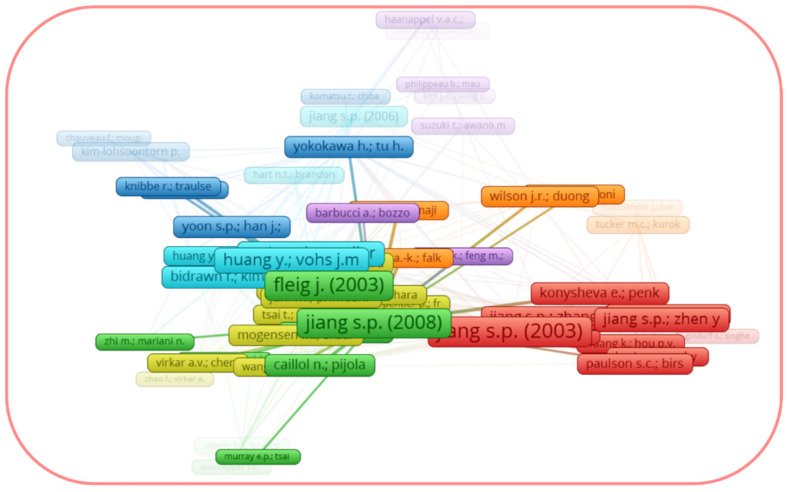
Network map of bibliographic coupling in the Scopus dataset for LSM.

**Figure 4:**
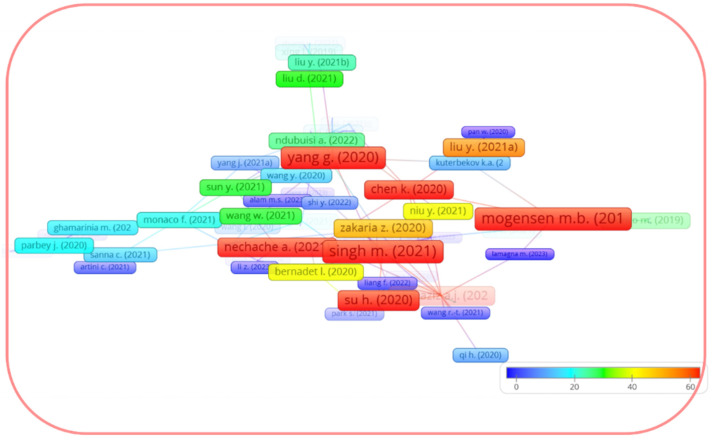
Network maps of bibliographic coupling in the Scopus dataset for cited documents of [33].

It is worth noting that the recent review by Chen et al. [54] presents the progress in surface modification techniques to improve the cathode performance, including methods such as solution infiltration, atomic layer deposition, and one-pot synthesis, but it does not directly address LSM and LSCF. In terms of LSM-related reviews, the recent review by Carda et al. [31], which highlights the mechanism of ORR on the LSM electrode, and the review by Jiang S.P. [30] on the development of the LSM cathodes, published in 2008, should be noted. Jiang’s review, which has 585 citations in the Scopus database as of June of 2023, can be found as the strongest item (excluding Jiang’s original research article [55]) in the bibliographic coupling link strength on the LSM network map, which is restricted to documents with at least 90 citations (Figure 3). In the case of LSCF, the reviews by Wang et al. [44], Jiang S.P. [33], and Safian et al. [36] (in chronological order) should be mentioned. The paper by Jiang [33], published in 2019, presents a comprehensive review of LSCF and has 223 citations in the Scopus dataset as of June of 2023. Figure 4 shows the network map of the bibliographic coupling in the Scopus dataset of documents citing the work [33] with their citations, considering a minimum number of citations of each citing document equal to five. The highest metrics of both the review by Jiang [33] and its citing documents show that the LSCF study topic is still highly relevant today. Meanwhile, the reviews [33,36,44], although considering LSCF, did not directly address the cathode enhancement strategies.

Therefore, in the present topical review we report on the most promising techniques to improve the electrochemical performance of conventional air electrodes for solid oxide fuel cells and also electrolysis cells based on the lanthanum strontium manganite La_1 − x_Sr_x_MnO_3 − δ_ with x = 0.2; 0.3; 0.4 as the commonly used compositions, and the lanthanum strontium cobaltite ferrite with the composition La_0.6_Sr_0.4_Co_0.2_Fe_0.8_O_3 − δ_ mainly, as the most prominent representative of the (La,Sr)(Co,Fe)O_3 − δ_ series and the most widely used cathode material for the intermediate-temperature (IT) solid oxide fuel cells (IT-SOFCs). The present work highlights for the first time the selection of optimal electrode compositions, as well as electrode fabrication and electrode activation methods.

## 2. Key Functional Properties of LSM and LSCF Electrode Materials: Advantages and Drawbacks

The lanthanum strontium manganite La_1 − x_Sr_x_MnO_3 − δ_ (LSM), as a representative of complex oxides with a perovskite ABO_3_ structure with rhombohedral distortions in the compositional range of 0.2 ≤ x < 0.4 [56,57,58], is known to be used as a material for the fabrication of air electrodes for electrochemical devices operating at low- [59] and intermediate temperatures [30]. On the one hand, this is due to the high level of electronic conductivity σ_e_ for LSM (corresponding to 200 S cm^−1^, 250 S cm^−1^, and 320 S cm^−1^ for La_0.8_Sr_0.2_MnO_3 − δ_ (LSM20), La_0.7_Sr_0.3_MnO_3 − δ_ (LSM30), and La_0.6_Sr_0.4_MnO_3 − δ_ (LSM40), respectively, at 800 °C and at Po_2_ = 1 bar [60]). Secondly, due to the closest values of the coefficient of linear thermal expansion (CTE) of the LSM (e.g., for LSM20—11.4 × 10^−6^ K^−1^ in the range of 50–1000 °C, [61]; for LSM30—12.2 × 10^−6^ K^−1^ and 13.2 × 10^−6^ K^−1^ in the ranges of 200–650 °C and 650–900 °C, respectively, [62]; LSM40—12.7 × 10^−6^ K^−1^ in the range of 50–1000 °C, [61];) to those for the solid electrolytes perspective for operating in IT-SOFCs and low-temperature SOFCs (LT-SOFCs) [15,22,24,63,64,65]. The CTE values can be mentioned for doped ceria (e.g., for Ce_0.8_Sm_0.2_O_2 − δ_ (SDC)—12.0 × 10^−6^ K^−1^ in the range of 25–1000 °C, [66]; for Ce_0.8_Gd_0.2_O_2 − δ_ (GDC)—12.2 × 10^−6^ K^−1^ in the range of 50–900 °C, [67]), (Sr,Mg)-doped LaGaO_3_ (in general LSGM, e.g., for La_0.8_Sr_0.2_Ga_0.8_Mg_0.2_O_3 − δ_—12.1 × 10^−6^ K^−1^ in the range of 25–1000 °C, [68]), Sc_2_O_3_-stabilized ZrO_2_ (SSZ) (for 8SSZ—10.4 × 10^−6^ K^−1^ in the range of 30–1000 °C, [69]). In addition, LSM electrodes offer such an important advantage as the improved stability during operation under SOFC and solid oxide electrolysis cell (SOEC) conditions compared to Fe- [48] and Co- [70,71] containing electrodes.

It should be noted that the oxygen diffusion and interfacial heteroexchange parameters for LSM, which is predominantly an electron conductor, are lower than those of materials possessing high mixed oxygen ion and electron conductivity (MIECs), such as cobalt-based perovskites. For example, the oxygen self-diffusion coefficient (D*) and the oxygen surface exchange coefficient (k) for LSM20 at 800 °C were found to be 4.00 × 10^−15^ cm^2^ s^−1^ and 5.62 × 10^−9^ cm s^−1^, respectively, compared to D* = 9.87 × 10^−10^ cm^2^ s^−1^ and k = 6.31 × 10^−7^ cm s^−1^ for the La_0.8_Sr_0.2_CoO_3 − δ_ MIEC material [72]. The values of the ionic conductivity, σ_i_, for LSM are in a range of 10^−4^–10^−7^ S cm^−1^ at 800–1000 °C and decrease significantly in the intermediate temperature range of 600–800 °C [30].

Recently Jiang summarized the literature data regarding the characterization of the oxide materials from the lanthanum strontium cobaltite ferrite (La,Sr)(Co,Fe)O_3 − δ_ series, and showed that the composition La_0.6_Sr_0.4_Co_0.2_Fe_0.8_O_3 − δ_ (the acronym LSCF will be used from now on), as a representative of MIECs, is the most prominent cathode material used in IT-SOFCs [33]. LSCF, which has a perovskite structure with rhombohedral distortions [73,74], exhibits excellent electrical properties properties with ionic and electron partial conductivity values reaching approximately 1 × 10^−2^ S cm^−1^ and 1 × 10^2^ S cm^−1^, respectively, at 800 °C [75]. It demonstrates a moderate CTE value equal to 17.5 × 10^−6^ K^−1^ in the range of 30–1000 °C [76]. The oxygen self-diffusion and surface exchange coefficients for LSFC amount 5 × 10^−7^ cm^2^ S^−1^ [77] and 6 × 10^−6^ cm S^−1^ [78] at 800 °C. It should be noted that due to MIEC conductivity nature and the superior oxygen diffusion properties of LSCF compared to LSM, this material is preferred for use in electrochemical devices operated at lower temperatures, whereas LSM materials are still in high demand for high-temperature applications due to their CTE values being more compatible with electrolyte materials and higher total conductivity values.

The main drawbacks of conventional perovskite electrodes, which lead to the degradation of solid oxide cells during long-term operation, are the segregation of Sr at the electrode surface with the formation of the SrO layer for the LSM- [35,52] and LSCF-based [36,79,80,81,82] cells, and the high interaction of LSM [41,52,83,84,85,86] and LSCF [41,87,88,89,90] with Zr-containing electrolytes to form the SrZrO_3_ (SZO) and La_2_Zr_2_O_7_ (LZO) phases. In addition, due to the presence of CO_2_ in the air, the SrCO_3_ carbonate phase was formed on the surfaces of the LSM- [91,92,93] and LSCF- [94,95,96] electrodes. The formation of the same insulating phases limits the oxygen exchange at the electrode–electrolyte interface and reduces the electrocatalytic activity of the LSM [97,98,99,100] and LSCF [79,100,101,102,103,104,105] air electrodes for the ORR, followed by an increase in both the electrode ohmic and polarization resistances. Similar long-term operation tests have shown that the degradation of cells based on LSM [81] or LSCF [106,107] operating in electrolysis mode was higher than that in fuel cell mode, and when comparing two perovskite electrodes, the long-term durability of LSCF was one step ahead of that of LSM [106,108].

Automated methods developed for the detection of Sr nucleation seeds on the electrode surface [109,110], for the identification of the interlayer width between the perovskite and YSZ layers [111,112], and for the computational design and numerical simulation of the perovskite-based composite electrodes [113,114,115,116] allowed for the suggestion of possible degradation mechanisms and an understanding of the electrode behavior at both the atomic and macroscopic scales. The deposition of protective and non-catalytic layers on the electrode surface has been proposed as a solution to the problems of segregation [117,118,119], contaminant poisoning [120,121,122,123], and sluggish oxygen kinetics [124,125,126,127,128]. The organization of ceria-based buffer layers at the perovskite electrode/YSZ electrolyte interface and the replacement of the zirconium electrolyte in the composite electrode with other ionic conductors, methods widely used for other perovskite electrodes [129,130,131,132,133,134,135], may also help to reduce interactions and increase the activity and long-term stability of LSM [136,137,138] and LSCF [138,139,140,141,142,143,144,145,146,147] electrodes. The high reactivity of the (La, Sr)-containing electrodes with the conventional YSZ electrolyte facilitated extensive investigations of the LSM-based cathodes formed on the SDC [148,149,150,151], GDC [152], SSZ [151,153], LSGM [149], BaZr_0.8_Y_0.2_O_3 − δ_ (BZY20) [154], La_9.5_Si_6_O_26.25_ [155], and La_27.44_W_4.56_O_55.68_ (LWO56) [156] electrolytes as an alternative. The LSCF-based cathodes were formed on the SSZ [157], SDC [158,159], Ce_0.9_Gd_0.1_O_2 − δ_ (GDC10) [160,161,162], GDC [126,163,164,165,166,167] Y_0.1_Ce_0.9_O_1.95_ [168], Nd_0.2_Ce_0.8_O_3 − δ_ [169], SSZ [170], LSGM [171,172], GDC with LSGM [173], SDC with LSGM [174], BZY20 [175], BaZr_0.9_Y_0.1_O_3 − δ_ [176], BaZr_0.8_Yb_0.2_O_3 − δ_ (BZYb) [177], BaCe_0.7_Zr_0.1_Y_0.1_Yb_0.1_O_3 − δ_ (BCZYYb) [178,179,180,181,182], BaCe_0.7_Zr_0.1_Y_0.2_O_3 − δ_ (BCZY) [183,184,185], and BaCe_0.7_Zr_0.15_Y_0.15_O_3 − δ_ (BCZY15) [186] electrolytes.

The chemical compatibility of the LSM and LSCF electrodes with oxygen-ion and proton-conducting electrolytes and the interdiffusion across the cathode/interlayer/electrolyte interfaces have been extensively observed in the recent reviews of Zhang et al. [41], Khan et al. [42] and Hanif et al. [187]. To briefly summarize the data presented in [41], it could be mentioned that LSM20, in contrast to LSCF, showed good chemical compatibility with La_27_W_4_NbO_55 − δ_ up to 1400 °C [188] and with La_10_Si_55_Al_0.5_O_26.75_ at 1300 °C [189], whereas the La-deficient LSM40 did not react with La_0.9_Sr_0.1_Ga_0.8_Mg_0.2_O_2.85_ (LSGM9182) at 1300 °C [190]. Secondary phases were found to form after sintering of LSM20 with SDC and BaCe_0.9_Y_0.1_O_3 − δ_ at 1150 °C [191], La-deficient LSM20 with BZY20 and BaCe_0.8_Y_0.2_O_3 − δ_ (BCY20) at 1100 °C [192], LSCF with BZY20 and BCY20 at 1100 °C [192]. The conclusions in [41] justified that LSCF had better chemical compatibility with LSGM and CeO_2_-based electrolytes due to the formation of small amounts of impurity phases with LSGM9182 at 1300 °C and negligible interdiffusion with SDC at 1150 °C.

It should be noted that the use of LSM electrodes in contact with the BaCeO_3_-based electrolytes is limited due to their active chemical interaction [193]. However, due to its high electronic conductivity and CTE compatibility, LSM can be successfully used as a collector for perspective layer electrodes for proton-conducting fuel cells operating in the IT range [194,195,196,197].

## 3. Conventional and Advanced Techniques to Fabricate Electrode Layers

**Figure 5:**
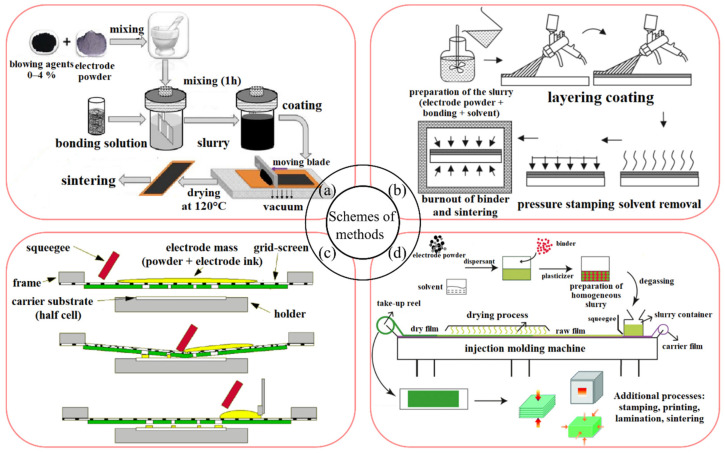
Schemes of methods used to apply electrode layers: (**a**) slurry application; (**b**) spraying; (**c**) screen printing; (**d**) film casting ((**a**) is reproduced from [225] with permission of IOP Publishing; (**b**) is reproduced from [226] with permission of Taylor and Francis; (**c**) is reproduced from [227]; (**d**) is reproduced from [228] with permission of J. Wiley and Sons).

The choice of electrode deposition method and electrode configuration usually depends on the cell design (tubular/planar configuration), which in turn determines the current characteristics of the electrode. Traditional ceramic methods of the oxygen electrode deposition in planar cells on the supporting fuel electrode, which are technologically simple and easily scalable, are slurry coating [198,199,200,201] (LSM), Refs. [202,203,204] (LSCF), screen printing [205,206,207,208,209] (LSM), Refs. [110,158,183,210,211] (LSCF), tape casting [212,213,214,215] (LSM), Refs. [174,180,216,217] (LSCF), spraying [212,218,219,220,221] (LSM), Refs. [170,173,222,223,224] (LSCF). Figure 5 shows schematics of these deposition methods.

In addition, freeze casting [229] (LSM), Refs. [230,231] (LSCF), dip coating [232,233] (LSM), Refs. [234,235,236,237,238] (LSCF), and extrusion [239,240,241] (LSM), Refs. [241,242,243,244] (LSCF) have been used to fabricate LSM- and LSCF-based electrodes in microtubular cells. Electrophoretic deposition [245,246] has been applied to form electrode thin films [247,248,249,250] (LSM), Refs. [251,252,253,254] (LSCF), and to fabricate LSM-based layered electrodes [255,256].

To maintain a high sintering temperature, e.g., to improve adhesion and/or to form air electrodes in the co-sintering process with NiO-based cermets with lower sinterability, different pore formers have been used to control the microstructure of the air electrode: carbon black [257,258,259,260,261] (LSM), Refs. [262,263,264,265] (LSCF); various types of starch [154,205,266,267,268,269] (LSM), Refs. [251,262,270,271,272] (LSCF).

To improve the catalytic activity of perovskite electrodes, special techniques for depositing nanoscale layers have been used, which are different from those used for the electrodes based on micro-sized electrode materials. In general, these are mainly the low-temperature methods to maintain the high surface area of the nanosized materials. Among the variety of methods available to improve the conventional perovskite electrodes by extending the triple-phase boundary (TPB), it is worth mentioning spray pyrolysis in its different modifications highlighted in the review [273], and used for LSM in [218,219,274,275,276,277], and for LSCF in [223,278,279,280,281]; electrospinning represented in reviews [282,283,284], and used for LSM in [285,286], and for LSCF in [286,287,288,289,290,291,292] template method used for LSM in [293], and for LSCF in [294,295,296,297]; infiltration method considered in the reviews [298,299,300,301,302], and used for LSM in [26,54,148,154,232,303,304,305,306,307,308,309,310,311,312], and for LSCF in [118,182,288,313,314,315,316,317,318,319,320,321,322,323,324,325,326,327,328,329,330,331,332,333,334,335,336,337,338,339,340,341,342,343,344]; solution combustion used for LSM in [345,346], and for LSCF in [346,347]. As a promising technique for the design of solid electrochemical cells, additive manufacturing (3D printing) has demonstrated its advantages for the fabrication of the scalable three-dimensional microstructures [348,349,350,351] using the LSM-based electrodes [352,353,354,355,356,357,358,359] and surface-modified LSCF electrodes [337,360,361].

**Figure 6:**
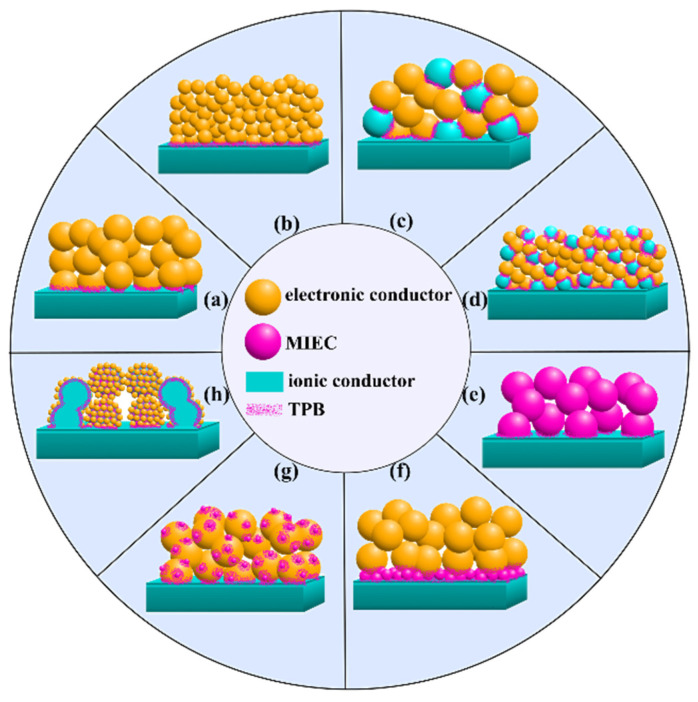
Air electrode activation method for electrochemical devices: (**a**) electronic conductor with localization of the TPB at the electrode–electrolyte interface; (**b**) nanosized electrode; (**c**) composite conductor/electronic conductor; (**d**) nanocomposite electrode; (**e**) mixed ionic-electronic conductor with extended TPB; (**f**) thin film active layer at the electrode–electrolyte interface; (**g**) electronic conductor in a porous ionic conductor matrix obtained by infiltration; (**h**) nanoparticle exfoliation on the surface of the electronic conductor.

Electrode activation methods are schematically summarized in Figure 6. They include the use of composite materials, materials with mixed electronic and ionic conductivity, the introduction of an active interlayer at the electrode–electrolyte interface (e.g., a highly conductive ionic conductor), the exfoliation of active nanoparticles on the electrode surface, infiltration, the use of nanomaterials. Due to the predominantly electronic nature of LSM materials, the selection and application of appropriate activation methods are necessary, especially for the electrodes used in electrochemical devices operating at decreased temperatures (below 800 °C).

## 4. Methods to Improve the Electrochemical Performance of the Conventional Electrodes

### 4.1. Optimization of the Oxide Composition

The interaction of LSM with Zr-containing electrolytes, as it was mentioned above, results in the formation of low conducting LZO and, at high strontium content, SZO phases at the electrode sintering stage at temperatures above 1000–1200 °C, depending on the material dispersity [85]. It has also been found that Mn readily dissolves in YSZ, leaving chemically active La_2_O_3_ at the LSM/YSZ interface, which reacts with ZrO_2_ to form LZO [114]. To reduce the interaction of LSM with Zr-containing electrolytes, the use of (LS)_x_M, a cation deficient in the A position, has been proposed [129]. It was found that the creation of the A-site deficiency suppressed the formation of the parasitic LZO phase due to the decrease in the La chemical potential. However, it should be noted that increasing the deficiency beyond a certain limit led to the release of the MnO_x_ phase in (LS)_x_M, resulting in the intensification of interphase reactions. Thus, recent studies on the interaction of (La_0.75_Sr_0.25_)_x_MnO_3 − δ_ (x = 1.0, 0.97, 0.95, 0.9 and 0.85) with the SSZ electrolyte showed that the optimum deficiency level was 0.95 [153]. The composite electrode (LS)_0.95_M containing 50 mol% SSZ was characterized by the lowest values of polarization resistance *R*_p_ (sometimes called *R*_η_, ASR in the works of different groups) and serial resistance *R*_s_ in the series studied, despite the high temperatures of the electrode formation. Therefore, the values of *R*_p_ and *R*_s_ for the electrodes sintered at 1200 and 1300 °C were equal to 1.21 and 1.94 Ω cm^2^ and 0.82 and 1.60 Ω cm^2^ at 950 °C, respectively.

The excess of Mn in La_0.85_Sr_0.15_Mn_y_O_3 − δ_ (LSMy, y = 1.1) was found to prevent the formation of chemically active La_2_O_3_ and thus, the formation of LZO. In [362], it was shown that the use of LSM1.1 instead of LSM in a composite electrode with 20 wt% YSZ resulted in a reduction of *R*_p_ from 1.6 to 0.6 Ω cm^2^ at 1000 °C. It has been shown that the modification of the YSZ electrolyte with Mn also results in a reduction of the LSM/YSZ interaction [363,364].

In the case of LSCF, the A-site cation-deficient materials with the (LS)_x_CF and L_x_SCF composition have been developed and widely used [175,365,366] due to their improved electrical conductivity and reduced CTE values [367]. Meanwhile, based on the results of the long-term stability study of the YSZ electrolyte-supported SOFC with the (LS)_0.98_SF cathode, Simner et al. [79] concluded that the high rate of the cell degradation (about 30% during 500 h) was mainly caused by Sr segregation at the cathode–electrolyte and cathode/collector layer interfaces, without any significant microstructural and chemical changes. Bucher et al. [366] also confirmed that L_0.96_SCF underwent strong Si-induced degradation at 600 °C in both wet and air atmospheres.

As a possible solution to the problem of the Sr segregation and contaminant poisoning in the perovskite electrodes, the use of high entropy oxides (HEOs) containing at least five co-dopants in an equimolar ratio in the A-position could be considered [19]. Recently, HEOs based on LSM have been reported, such as La_0.2_Pr_0.2_Nd_0.2_Sm_0.2_Sr_0.2_MnO_3_ [368] and La_0.2_Nd_0.2_Sm_0.2_Ca_0.2_Sr_0.2_MnO_3_ [369]. It was shown that the distortions in the LSM perovskite structure caused by the multi-doping in the A-site led to the highly disordered stress field around the Sr ions, which resulted in limiting the transport and migration of the Sr ions, and suppressed the Sr segregation phenomenon [368,369]. Dabrowa et al. [370] synthesized a nanosized homogeneous high entropy oxide (HEO) based on LSCF, such as La_0.6_Sr_0.4_Co_0.2_Cr_0.2_Fe_0.2_Mn_0.2_Ni_0.2_O_3 − δ_. The resulting HEO was characterized by lower CTE, increased electronic conductivity, and enhanced stability to Cr contamination compared to the base oxide [370].

Xu et al. [371] synthesized the orthorhombic phase La_0.2_Nd_0.2_Gd_0.2_Sr_0.2_Ba_0.2_Co_0.2_Fe_0.8_O_3 − δ_ (HE–LSCF) and investigated the electrochemical activity of the HE–LSCF on the SDC substrate in the symmetrical cells. The *R*_p_ value for the HE–LSCF cathode was measured to be 0.57 Ω cm^2^ at 700 °C compared to 0.71 Ω cm^2^ for the conventional LSCF cathode. The kinetics of the oxygen reduction reaction in the HE–LSCF cathode, related to the processes of oxygen dissociation and interfacial charge transfer, was attributed by the authors to the middle-frequency process. The polarization results obtained for the cells aged at 800 °C for 200 h showed that the HE–LSCF cathode was characterized by higher stability compared to the pristine LSCF cathode. The *R*_p_ values were 0.75 and 1.27 Ω cm^2^ at 700 °C for the high-entropy LSCF cathode and the conventional LSCF cathode, respectively. Scanning electron microscopy (SEM) and energy dispersive spectroscopy (EDS) data confirmed that the surface of the HE–LSCF cathode had a homogeneous distribution of all elements in the A-position with no Sr segregation both before and after cell ageing in contrast to the pristine LSCF cathode. The authors reported that the Sr segregation was successfully suppressed in the HE–LSCF cathode.

In summary, although the formation of the (La,Sr)-deficient sites, which aims at both increasing the electrical conductivity and suppressing Sr segregation is the most widely used technique for selecting the optimal composition of the LSM and LSCF electrodes, the creation of highly disordered oxides with sluggish cation diffusion can be quite perspective direction in the composition modification of the conventional perovskite materials.

### 4.2. Enhancement of the Ionic-Conducting Electrode Component

To increase the electrochemical activity of LSM air electrodes, their compositions with ionic conductors were applied, e.g., with solid electrolytes (YSZ [205,303,352,372,373,374,375,376,377,378,379,380,381,382,383], YSZ with GDC10 [374], SSZ [149,384,385,386,387,388,389,390], GDC10 [391], GDC [152,198], SDC [148,149,387,392,393], doped Bi_2_O_3_ [151,394,395,396,397,398,399], BaZr_0.85_Y_0.15_O_3 − δ_ (BZY15) [154], LWO56 [156], LSGM [400,401]), or MIEC materials (Co- [150,402] and Fe-based perovskites [403,404,405]). The optimum electrolyte content in the composite electrode depends on the specific surface area ratio of the components and is in the range of 30–50 wt% in most cases. Calculations using *Comsol* software showed that the maximum active TPB of 9.53 µm μm^−3^ was achieved for electrodes containing 35 vol% of the electrode material, 35 vol% of the electrolyte and 30 vol% of pores [396].

Wang et al. [373] found that the enhancement of the electrochemical activity of the LSM–YSZ composites was due to the spatial enlargement of the triple-phase boundary (TPB) area, which increased the active sites for the oxygen adsorption and charge transfer processes. Lee et al. [383] reported on dissociative adsorption as the main rate-determining step of the ORR on the LSM–YSZ composite cathodes. It was found that the replacement of YSZ with GDC in the LSM–based composite electrodes developed in [152] resulted in much lower current interfacial resistances compared to the conventional LSM–YSZ electrodes on the corresponding electrolytes.

**Figure 7:**
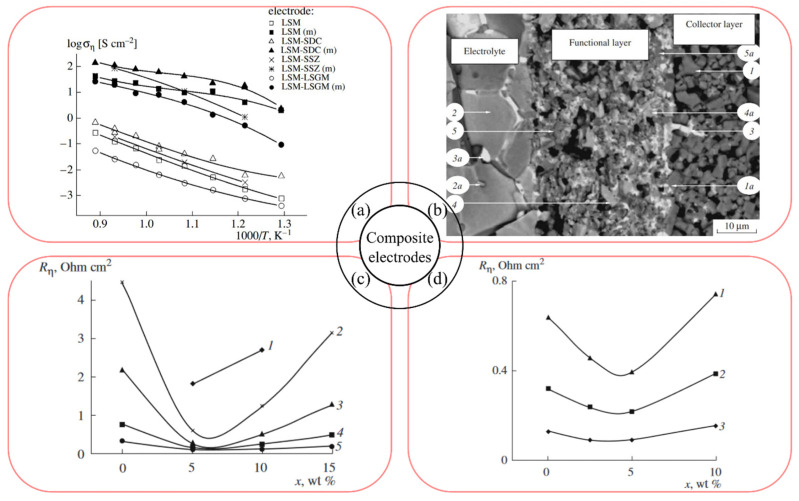
Effect of activation on the properties of composite electrodes of different composition applied by screen printing on SDC electrolyte: (**a**) temperature dependences of polarization conductivity for basic and praseodymium oxide (m) modified 50 wt% LSM20 based electrodes; (**b**) SEM micrograph showing diffusion of bismuth-based fusible components from 95LSM40–5YDB collector into 50LSM20–50SDC functional layer and YSZ electrolyte (Bi-containing phases are denoted by 3, 3a and 5, 5a); (**c**,**d**) polarization resistance of 50LSM20–50SDC and 50LSM20–50ScSZ electrodes with LSM40 collector as a function of its YDB content (*x*) ((**a**) is reproduced from [149] with permission of Springer; (**b**–**d**) are reproduced from [151] with permission of Springer).

A detailed study of the influence of ionic conductivity on the properties of the LSM20-based composite electrodes was carried out by Yaroslavtsev et al. in [149], using high ionic conductivity materials SSZ, SDC and La_0.88_Sr_0.12_Ga_0.82_Mg_0.18_O_2.85_ (LSGM8282) as the electrolyte component, whose content in the composite electrodes was 50 wt%. The thickness of the electrodes, obtained by screen-printing of a powder suspension with polyvinyl butyral (PVB) binder with the addition of ethyl alcohol, was 50 µm. A collector layer of LSM40 composition was used for the composite electrode to ensure stable current collection. Sintering of the 50LSM20–50SSZ micro-sized electrode layers was carried out at 1200 and 1250 °C, 1 h. Figure 7a shows the characteristics of the electrodes after sintering and after activation with praseodymium oxide (m) in symmetrical cells based on the Sm-doped ceria electrolyte of the composition often used to organize buffer layers at the air electrode–electrolyte interface in ZrO_2_ and LaGaO_3_-based cells). The 50LSM20–50SDC composite electrode was found to have the best performance, with the 50LSM20–50SSZ electrode with LSM40 collector coming close. The polarization conductivities of the modified 50LSM20–50SDC and 50LSM20–50SSZ/LSM40 electrodes were 114 and 100 S cm^−2^ at 800 °C, corresponding to *R*_p_ of 0.009 and 0.01 Ω cm^2^.

Furthermore, Yaroslavtsev et al. have proposed to modify the properties of 50LSM20–50SDC and 50LSM20–50SSZ electrodes by activation through the LSM40 collector layer containing Y-doped bismuth oxide Bi_1.5_Y_0.5_O_3 − δ_ (YDB) [151]. It was shown that when the collector layer was sintered at 1000 °C, the polarization resistance of the electrodes decreased due to the diffusion of bismuth-containing melt phases into the functional composite layer (Figure 7b). The optimum additive content in the collector layer for both electrodes was therefore set at the level of 5 wt% (Figure 7c,d). When the YDB content was increased beyond this value, a decrease in the cathode performance was observed due to the sintering and reduction in porosity of the collector layer. The *R*_p_ values of 0.1 and 0.2 Ω cm^2^ at 800 °C were obtained for the 50LSM20–50SDC and 50LSM20–50SSZ electrodes, which were optimized in terms of sintering temperature and amount of the additive. The composite electrodes, activated through the collector, exhibited higher *R*_p_ values compared to the praseodymium oxide-activated electrodes; however, they demonstrated a significantly lower degradation rate.

Mosiałek et al. [150] investigated the performance of the composite cathodes containing LSM20 and the MIEC material of YFe_0.5_Co_0.5_O_3_ (YFC) formed on the SDC electrolyte by screenprinting. It was found that the addition of 5 wt% YFC to LSM resulted in a decrease of the polarization resistance *R*_p_ from 2.7 to 1.9 Ω cm^2^ at 800 °C. This fact was attributed to the facilitation of the charge transfer process.

LSM composite electrodes can be prepared by various techniques: by mechanically mixing the components in a mill [114,396], by forming composites with a core–shell structure (by depositing one material on top of another) [206], by infiltrating one component of the composite electrode into the pre-sintered porous matrix of the other component [198,377], and by preparing vertically oriented high-entropy nanocomposites [393].

In [206], the 60(LS)_0.95_M25–40YSZ composite powder was obtained by the synthesizing of LSM on submicron YSZ particles using in situ glycine-nitrate combustion method. The electrode with a core–shell structure was formed on the surface of the YSZ electrolyte by screen printing (Cell A). The organic binder consisted of 5 wt% ethyl cellulose dissolved in 95 wt% terpineol, and the weight ratio of electrode powder to the binder in the stencil was 2:3. The applied electrode was sintered at 1180 °C for 2 h. For comparison, the second electrode was formed under the same conditions from a mechanical mixture of YSZ and LSM, synthesized by the glycine-nitrate combustion method (Cell B). The YSZ electrolyte formation in the half-cells on the NiO–YSZ support cathode for the single-cell test was also carried out by screen printing with sintering at 1400 °C for 2 h, with a film thickness of about 10 μm. The authors observed a reduction in the electrolysis cell performance when using an anode produced by conventional mixing. Current densities of 0.520 A cm^−2^ and 0.431 A cm^−2^ at 900 °C and 0.333 A cm^−2^ and 0.231 A cm^−2^ at 850 °C at 1.50 V were obtained for Cell A and Cell B, respectively.

**Figure 8:**
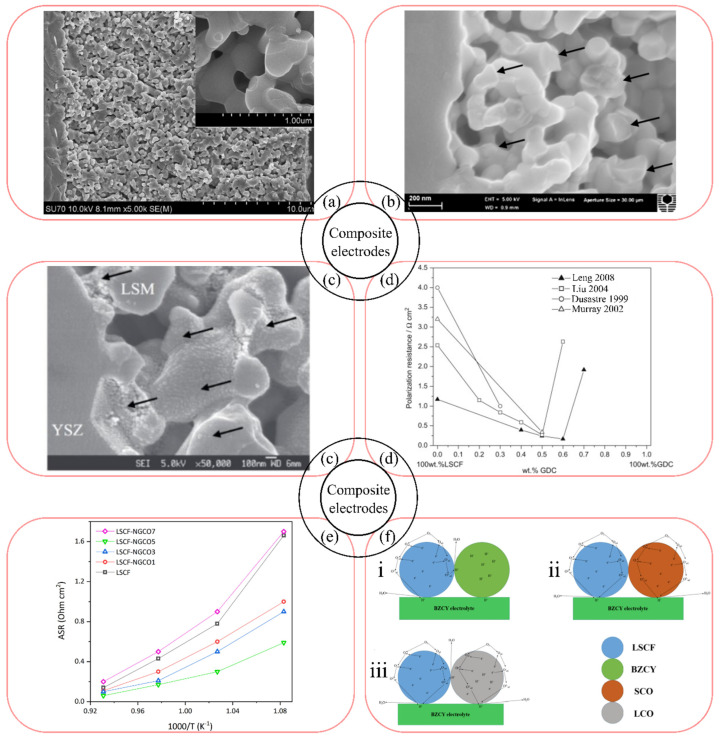
SEM micrograph of the 50(LS)_0.95_M25–50YSZ electrode of 20 µm thickness, prepared by nanopowder suspension spraying followed by sintering at 1000 °C, 2 h (**a**); YSZ ceramic matrix followed by heat treatment at 600 and 1100 °C (arrows indicate LSM particles) (**b**); SEM micrograph of the 75LSM20–25GDC electrode obtained by GDC infiltration into the LSM ceramic matrix (arrows indicate GDC particles) (**c**); polarization resistance of the LSCF–GDC10 composite cathodes on the GDC10 content at 600 °C by Leng et al. [407], Liu et al. [408], Dusastre et al. [409], Murray et al. [410], (**d**); temperature dependencies of the polarization resistance for LSCF–NGCO cathodes characterized in symmetrical cells (**e**); scheme of migration paths of conducting species in LSCF–BZCY (**i**), LSCF–SCO (**ii**), LSCF–LCO (**iii**) composite cathodes on BZCY electrolyte (**f**) ((**a**) is reproduced from [378] with permission of Elsevier; ((**b**) is reproduced from [377] with permission of Elsevier; (**c**) is reproduced from [198] with permission of Elsevier (**d**) is reproduced from [407] with permission of Elsevier; (**e**) is reproduced from [166]; (**f**) is reproduced from [185] with permission of Elsevier).

It should be noted that the dispersity of the materials used has a great influence on the performance of composite electrodes obtained by mechanical mixing. It is therefore important to obtain LSM powders with nanosized particles. According to the data reported in [406], La_0.5_Sr_0.5_MnO_3 − δ_ (LSM50) with homogeneous morphology and nanoparticles of 20 nm, prepared by the Pechini method, has been characterized as a material that provides improved electrode activity compared to those obtained with LSM50 powders synthesized by citrate and alkoxide techniques. The application of nanosized (86 nm) low-agglomerated powder (La_0.75_Sr_0.25_)_0.95_MnO_3 − δ_ ((LS)_0.95_M25), obtained by pyrolysis of acetate acrylic polymer, in the composite with YSZ reduced the electrode polarization to less than 0.1 Ω cm^2^ at 800 °C [378]. A 20 µm thick 50(LS)_0.95_M25–50YSZ composite was formed in a NiO–YSZ|YSZ half-cell by spraying an ethanol-based suspension followed by drying and sintering at 1000 °C for 2 h (Figure 8a).

The application of infiltration leads to both an increase in the activity of the composite electrode and a solution to the problem of electrode delamination, which is particularly pronounced under conditions of anodic polarization. For example, the 45LSM20–55YSZ nanostructured electrodes, obtained in [377] by infiltration of La_0.8_Sr_0.2_Mn(NO_3_)_x_ with addition of citric acid into the porous YSZ ceramic matrix (Figure 8b), followed by a two-step temperature treatment at 600 °C and then at 900 or 1100 °C, showed *R*_p_ values of 0.21 and 0.74 Ω cm^2^ at 800 °C, respectively. The YSZ matrix for the composite electrode was formed in [377] on the YSZ electrolyte surface by slurry coating followed by sintering at 1200 °C for 2 h. In [198], the 75LSM20–25GDC composite was prepared by infiltrating the solution of Gd_0.2_Ce_0.8_(NO_3_)_x_ into a 20–30 µm-thick porous LSM matrix (Figure 8c) formed by slurry deposition followed by presintering at 1100 °C for 2 h. The polarization resistance of the bare LSM electrode measured at 8.2 Ω cm^2^ at 800 °C was reduced to 0.39 and 0.09 Ω cm^2^ after infiltration with 0.5 and 1.5 mg cm^−2^ GDC, respectively. The electrodes, developed by Chen et al. [198,377], showed high stability under polarization with an anodic current of 500 mA cm^−2^ at 800 °C in air for 100 h. However, it should be noted that the area of the infiltrated electrodes is generally small (<1 cm^2^) because it is difficult to obtain a homogeneous distribution of the component in the matrix over a large area in a non-mechanized infiltration process.

For the preparation of the LSCF-based electrodes, compositions with ionic conductors were used, such as YSZ [411,412], YSZ with GDC10 [413], GDC10 [160,336,407,414,415,416,417,418] GDC [110,120,171,417,418,419,420,421], Nd_x_Gd_0.15_Ce_0.85 − x_O_2 − δ_ (NGCOx) [166], SDC [114,127,162,184,185,416,422,423,424,425,426], Ce_0.8_Zr_0.2_O_2 − δ_ [427], BZYb [177], BCZYYb [178,181], BCZY [185], BCZY15 [186], La_2_Ce_2_O_7_ (LCO) [185], LSGM [428,429], as well as with the MIEC materials Bi_0.3_Sr_0.7_Co_0.3_Fe_0.7_O_3 − δ_ [172], La_0.94_Ni_0.6_Fe_0.4_O_3_ (LNF94) [340], Ba_0.5_Sr_0.5_Co_0.8_Fe_0.2_O_3 − δ_ [430], SrCo_0.2_Fe_0.6_Ni_0.2_O_3 − δ_ [431]. As in the case of the LSM-based composites, the LSCF-based composite electrodes can be prepared by conventional mixing [166,185,407], infiltration [340,416,417,418,421,425,426], and by creating core–shell structures [340,424].

Ling et al. [407] evaluated the electrochemical activity of the pristine LSCF cathode and the LSCF–GDC10 composite cathodes at the operating temperatures of 500–700 °C. The *R*_p_ value of the LSCF porous electrode, sintered on the thin-film GDC10 electrolyte at 975 °C, was measured to be 1.20 Ω cm^2^ at 600 °C. The GDC10 additives to LSCF improved the electrode performance, reducing the polarization resistance down to 0.17 Ω cm^2^ at 600 °C for the LSCF–GDC composite cathode with the GDC10 content of 60 wt%. The higher GDC10 content resulted in an increase of polarization resistance, which was consistent with the ambipolar resistivity model of the porous composite cathode [409]. It has been shown that the polarization resistance increases in the composites with high electrolyte content because the electron-conducting pathways cannot be effectively formed [407]. Besides, both the ohmic resistance and the contact resistance were high for the LSCF–GDC10 composite cathode with the GDC10 content of 70 wt%. Therefore, it was concluded that the optimum GDC content to achieve the lowest polarization resistance of the LSCF–GDC10 composite cathode was 60 wt%. The performance of the single cell with the optimized LSCF–GDC composite as the cathode, GDC10 as the electrolyte, and Ni–GDC10 as the anode was characterized by the maximum power density (MPD) value of 422 mW cm^−2^ at 600 °C. The superior electrochemical performance of the LSCF–GDC10 composite cathode, observed in [407], compared to that obtained in [408,409,410] (as shown in Figure 8d) is consistent with the model that the optimum GDC10 volume fraction to achieve the minimum polarization resistance may be greater than 50 wt%, since the grain size of the LSCF powder is much smaller compared to that of the GDC powder.

A simple method for the preparation of nanosized composites has been proposed by Xi et al. [415]. The LSCF–GDC10 powders were obtained from La_2_O_3_, Sr(OH)_2_, Co_3_O_4_, Fe_2_O_3_, and GDC10 powders, mechanically treated in a high-speed attrition-type mill for 20 min. The resulting composite electrodes, sintered on the SSZ substrate at 900 °C, were characterized by the corresponding polarization resistance value of 0.38 Ω cm^2^ at 700 °C. The authors of [415] concluded that the mechanical treatment allowed the formation of nanosized particles in the LSCF–GDC10 composite as compared to the conventional mixing technique with the formation of micro-sized particles.

Samreen et al. [166] used the co-doped GDC for the fabrication of the LSCF–NGCOx composite electrodes (x was equal to 1, 3, 5, 7 wt%), obtained from the corresponding powder mixtures. According to the electrochemical impedance spectroscopy (EIS) data obtained on the symmetrical cells with the LSCF–NGCOx electrodes, sintered at 1100 °C on the GDC substrate, the lowest *R*_p_ equal to 0.31 Ω cm^2^ at 700 °C was observed for NGCO5. As shown in Figure 8e, the NGCO5-based electrode showed better performance not only compared to LSCF and other studied LSCF–NGCOx electrodes [166], but also compared to the LSCF–GDC10 electrodes [415]. Thus, the Nd co-doping of GDC can be considered as the promising way for the fabrication of the composite electrodes based on LSCF due to the improvement of the oxygen ion diffusion pathways and the optimization of the TPB sites.

In [120], the GDC-coated LSCF composite cathode prepared by the dip-coating technique was shown to have both the excellent electrochemical performance and the Cr poisoning tolerance. According to the EIS results, the 76LSCF–24GDC composite cathode was characterized by the polarization resistance value of 0.59 Ω cm^2^ at 700 °C, compared to 0.87 Ω cm^2^ at 700 °C for the bare LSCF. The 76LSCF–24GDC and LSCF cathodes showed the *R*_p_ values of 0.71 and 1.35 Ω cm^2^ at 700 °C, respectively. after the stability tests under the Cr poisoning conditions for 200 h. Zhang et al. [120] considered that the Cr poisoning of the composite cathode was reduced due to the diminishing of both Cr deposition on the electrode surface and the SrCrO_4_ formation.

Three different types of the LSCF-based composite cathodes for proton-conducting fuel cells (PCFCs) were obtained and investigated in [185]. The LSCF–SDC, LSCF–BCZY, LSCF–LCO electrode slurries were prepared from the LSCF and SDC, BCZY, and LCO powders, respectively, mixed in a 7:3 weight ratio using terpineol and ethyl cellulose as binders. The power densities of the anode-supported cells (ASC) with the LSCF–SDC, LSCF–BCZY, and LSCF–LCO cathodes, sintered on the BCZY electrolyte at 1000 °C, were measured to be 421, 432, 469 mW·cm^−2^ at 600 °C. The long-term operation tests of the single cell with the LSCF–LCO cathode showed the stability of the cell power of 420 mW cm^−2^ during 100 h at 600 °C without any obvious degradation. Gao et al. [185] suggested that the excellent performance of the LSCF–LCO cathode, compared to LSCF–SDC and LSCF–BCZY was due to the enhanced migration rates of proton and oxygen ions through the BCZY electrolyte, as shown in Figure 8f.

According to the conclusions of the work [421], the LSCF-based cathodes, obtained as a three-dimensional network of nanofibers had such advantages as high porosity, high percolation, continuous paths for charge migration, good thermal stability, and excellent framework for subsequent infiltration. The maximum power densities of the single cells with the NiO–YSZ as anode, YSZ as electrolyte, LSCF nanofiber cathode, prepared by electrospinning, and LSCF–GDC composite, prepared by the GDC infiltration, were measured to be 900 and 1070 W cm^−2^ at 1.9 A cm^−2^ at 750 °C, respectively. The polarization resistances of the LSCF and LSCF–GDC composite cathodes were equal to 0.26 and 0.21 Ω cm^2^ at 750 °C, respectively. In [417], the LSCF–GDC10 composite cathodes were formed from the LSCF nanofibers deposited on the GDC10 electrolyte, and the GDC10 was introduced into the LSCF scaffold by the infiltration method. The polarization resistances for the LSCF nanofiber and LSCF–GDC10 (weight ratio of 1:0.56) composite cathodes were equal to 0.78 and 0.14 Ω cm^2^ at 700 °C, respectively. The size of the infiltrated particles also may have an influence on the electrode performance. Burye and Nicholas [432] used desiccation of LSCF precursor nitrate solutions infiltrated into porous GDC10 scaffold with CaCl_2_ to decrease the average size of the infiltrated LSCF particles in the LSCF–GDC10 composite cathode down to 22 nm, which was more than twice lower than that in the pristine cathode. It allowed polarization resistance of 0.10 Ω cm^2^ to be obtained at 575 °C, compared to 650 °C for the undesiccated electrode. The results of the works [417,421,432] demonstrated the efficiency of the infiltration method in achieving the high cell performance of the solid oxide fuel cells.

The 50LSCF–50SDC core–shell composite cathode was prepared in [424]. The 500-nm size of the SDC core was controlled to achieve the total encapsulation of the SDC particles with the LSCF particles, resulting in the improved phase homogeneity with the excellent microstructure and increased TPB. The LSCF–SDC core–shell cathode was characterized by a polarization resistance of 0.265 Ω cm^2^ at 650 °C and long-term operational stability during both the 120 h electrochemical test and the 30 thermocycles between 100 and 650 °C [424]. According to the data of [340], the LNF94-infiltrated LSCF composite cathode with the core–shell structure on the GDC10 electrolyte was characterized by the values of *R*_p_ equal to 0.041 Ω cm^2^ and MPD, equal to 1080 mW cm^−2^ at 800 °C, and the excellent long-term stability in CO_2_ and Cr-containing atmospheres. The authors [340] showed that it was the heterogeneous electrode interface that significantly increased the electron conductivity and the oxygen dissociation.

Wang et al. [433] proposed a novel core–shell LSCF-based perovskite structured electrocatalyst covered with Ruddlesden–Popper phase La_0.6_Sr_1.4_Co_0.2_Fe_0.8_O_4 − δ_ (LSCF_214_) thin film. To form a local LSCF_214_ structure on the LSCF particles, Sr(NO_3_)_2_ was added to the suspension of LSCF particles simultaneously with urea as a complexing agent. After suspension evaporation on a hot plate, a uniform Sr^2+^ coated LSCF particle precursor was obtained, which was finally calcined at 800 °C. The core–shell electrode formed by screen-printing followed by sintering at 900 °C, 2 h on the SDC electrolyte demonstrated a lower polarization resistance (0.17 Ω cm^2^) than the LSCF electrode (0.32 Ω cm^2^) at 650 °C. The MPD values obtained for the anode-supported cell with the thin-film SDC electrolyte and the core–shell and conventional LSCF electrodes were 0.57 and 0.3 W cm^−2^ at 650 °C, respectively. Moreover, the core–shell electrode showed a low degradation rate in the long-term test at 600 °C. The ASR values at 0, 100, 200, 300 and 400 h were 0.58, 0.77, 0.84, 0.85 and 0.82 Ω cm^2^, respectively.

In summary, the preparation of the composite electrodes, aimed at increasing the TPB by improving the oxygen ion migration, remains the most demanded strategy to enhance the electrochemical performance of the traditional perovskite electrodes. However, the latest composite fabrication techniques use nanostructured materials to provide an increased electrode interface.

### 4.3. Improvement of the Electrode Surface

The effective approach to increase the electrochemical activity of the conventional perovskite electrodes by increasing the TPB is related to the preparation of decorated electrodes by the infiltration (also called impregnation) method, where the porous cathode is filled with various additives [148,163,304,323,340,382,403,434,435,436,437,438] as well as decorating the electrode surface with nanocatalysts [141,154,315,387,405,416,426,439] and nanocoatings [303,308,341,342], which significantly improve the electrode surface diffusion and exchange with the gas phase.

The enhancement of the electrochemical performance of (La_0.8_Sr_0.2_)_0.95_MnO_3 − δ_ ((LSM20_0.95_)–YSZ cathodes by infiltrating salt solutions was reported in [382]. The developed 50LSM20_0.95_–50YSZ cathodes were characterized by the reduction of the high-frequency polarization resistance up to 45% of the baseline (*R*_p(hf)_ = 1.06 Ω cm^2^ at 700 °C for the YSZ infiltrated electrode), and the low-frequency polarization resistance up to 28% of the baseline (*R*_p(lf)_ = 0.57 Ω cm^2^ at 700 °C for the ammonium chloride infiltrated electrode).

According to Zhang et al. [304] and Han et al. [305], the reversible cell with the LSM20–YSZ air electrode infiltrated with SrTi_0.3_Fe_0.6_Co_0.1_O_3 − δ_ (STFC), the YSZ electrolyte, and the SrTi_0.3_Fe_0.7_O_3 − δ_ fuel electrode showed the improved power performance using different fuel gases. The STFC infiltration allowed for an increase in the peak power density of the solid oxide cell in the fuel mode by >1.5 times, up to 0.88 W cm^−2^ and 1.37 W cm^−2^ using air and oxygen as an oxidizer, respectively [304], and up to 0.9 W cm^−2^ and 0.68 W cm^−2^ using wet H_2_ and wet CO as a fuel, respectively [305], at 800 °C.

**Figure 9:**
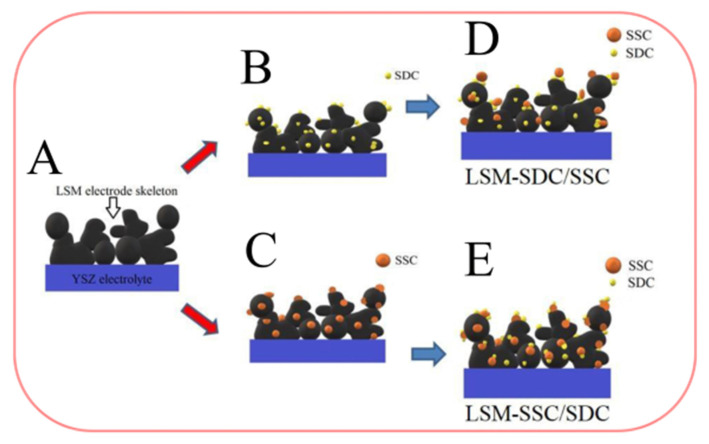
The schemes for the alternate infiltration: LSM electrode (**A**), SDC infiltrated LSM (**B**), SSC infiltrated LSM (**C**), SDC and SSC alternately infiltrated LSM (**D**), SSC and SDC alternately infiltrated LSM (**E**) (reproduced from [148] with permission of Elsevier).

Wu et al. [148], who investigated the influence of the infiltration process on the morphology and performance of the LSM electrode (electrode schemes are presented in Figure 9), showed that the electrochemical characteristics of the LSM–SDC electrodes infiltrated with Sm_0.5_Sr_0.5_CoO_3 − δ_ (SSC) and the LSM–SSC electrodes infiltrated with SDC (LSM–SSC–SDC) were dependent on the infiltration time. It was shown that as the infiltration time increased, the cathode polarization resistance and overpotential decreased, and the single-cell peak power density improved. The *R*_p_ value for the LSM–SSC–SDC electrode was obtained as low as 0.08 Ω cm^2^ at 800 °C compared to 2.38 Ω cm^2^ at 800 °C for the LSM electrode.

Sun et al. in [154] developed the composite electrode for a protonic ceramic fuel cell based on (LS)_0.95_M20 and BZY15 infiltrated with Pr_6_O_11_ nanoparticles over four cycles. The polarization resistance of the (LS)_0.95_M20–BZY15 cathode on the BZY20 electrolyte, measured at 600 °C, was equal to 0.12 Ω cm^2^, and the peak power density of the NiO–BZY20|BZY20|LSM–BZY15–Pr_6_O_11_ single cell reached 0.28 W cm^−2^ at 600 °C. The long-term stability tests showed that the obtained cathode was stable at 600 °C for 100 h. The results of the work [154] demonstrated that the infiltration technique, which ultimately led to the TPB extension, is a highly effective strategy to improve the electrochemical activity of conventional LSM–YSZ cathodes by enhancing the oxygen adsorption, dissociation, and diffusion processes.

In [387], the possibility of using the low-cost mixed metal LnO_x_ (Ln = Ce, La, Nd, Pr, Sm) as an ionic conductor in an air nanostructured electrode was demonstrated, and the *R*_p_ value, equal to 0.04 Ω cm^2^ at 800 °C, was measured for the composite electrode of praseodymium oxide-modified 60LSM20–40LnO_x_. The influence of the cerium, praseodymium or manganese oxide infiltration on the electrochemical performance of the 80(LS)_0.95_M20–20YSZ electrodes was investigated in [306]. It was shown that the infiltration of oxide nanoparticles significantly increased the electrochemical performance of LSM–YSZ: infiltration of PrOx resulted in a polarization resistance value of 0.068 Ω cm^2^ at 700 °C.

Seyed-Vakili et al. [405] investigated the effect of co-infiltration with metallic (Ag) and ceramic (La_0.8_Sr_0.2_FeO_3_ (LSF20), CeO_2_) precursors on the performance of the LSM cathode on the YSZ substrate. It was shown that the introduction of Ag and ceria reduced the polarization resistance down to 0.64 Ω cm^2^ at 800 °C, which was 2.5% lower than that of the pristine LSM electrode, while the infiltration of Ag solution reduced the electrode overpotential by approximately 114%.

The results of the study [303] showed that the enhancement of the LSM20–YSZ electrode activity can be achieved by using a nanocoating consisting of the dual (CoOx, Pt) nanocatalyst. The polarization resistance values for the electrodes without and with deposited nanocatalysts were equal to 0.66 and 0.31 Ω cm^2^ at 750 °C, respectively. In addition, the long-term operation of the NiO–YSZ|YSZ|LSM20–YSZ single cell at 750 °C showed that the use of the nanocoating resulted in an increase in the MPD value of almost 200%.

The improved electrochemical performance of the LSM20 cathode on the YSZ electrolyte substrate was achieved by depositing the nanocrystalline YSZ interlayer at the LSM/YSZ interface using an infiltration process [308]. The LSM–YSZ infiltrated cathode showed a reduced polarization resistance value of 0.11 Ω cm^2^ compared to 0.35 Ω cm^2^ for the LSM infiltrated cathode (at 750 °C). In addition, the single cell NiO–YSZ|YSZ|LSM20 with the nanocrystalline YSZ interlayer was characterized by the increased peak power density at 750 °C, equal to 1.54 W cm^−2^, compared to the value of 0.76 W cm^−2^ for the cell without the YSZ interlayer. The single cell based on the LSM–YSZ infiltrated cathode showed long-term stability at 750 °C for 300 h at a current density of 0.5 A cm^−2^. Koo et al. attributed the improved oxygen reduction kinetics to the increased TPB on the infiltrated YSZ interlayer, which increased the number of reaction sites with a low reaction barrier [308].

The LSCF–GDC10 composite electrodes decorated with CuO nanoparticles were prepared by the infiltration technique in [416]. The results of the high-temperature X-ray diffraction study indicated the formation of a new Cu-containing LSCF-based compound. The polarization resistance of the LSCF–GDC10 cathode on the GDC10 substrate decreased significantly from 0.62 Ω cm^2^ to 0.32 Ω cm^2^ at 650 °C for the bare and infiltrated electrodes, respectively. Gao et al. concluded that the electrode improvement was due to the Cu^2+^ reduction at the LSCF–GDC10/CuO interface. Meanwhile, a slight degradation in the performance of the infiltrated samples was observed during the ageing tests at 500 °C and 650 °C for 150 h in air. The authors of [416] attributed the electrode degradation to the coarsening of the CuO particles, the Sr segregation and the low stability of Cu-containing impurities formed at the interface.

The 50LSCF–50Er_0.4_Bi_1.6_O_3_ (ESB) composite cathode prepared by infiltration using organic solvents in [323] showed the excellent electrochemical performance with the SDC electrolyte, equal to 0.11 Ω cm^2^ at 700 °C, compared to 0.27 Ω cm^2^ at 700 °C for the LSCF cathode. It was also found that this value was the best performance for the LSCF infiltrated electrodes reported in the literature, such as LSCF–Ce(Ag) [315], LSCF–(Pr,Ni,Mn)O [341], LSCF–Pd [439], LSCF–LSM [403], LSCF–SDC [425], LSCF–SDC–CuO [416], LSCF–SDC–Ag [426], LSCF–GDC10 [417,418]. The NiO–SDC|SDC|LSCF–ESB single cell fuelled with wet hydrogen, characterized by an MPD value of 469 mW cm^−2^ at 700 °C had a good short-term stability for 50 h at a current density of 0.45 A cm^−2^ [323]. Wang et al. concluded that the ESB additive had changed the rate-limiting electrode process from the charge transfer to the oxygen diffusion on the cathode surface.

Shi et al. [341] reported on the enhanced electrochemical activity of the LSCF cathode by coating a thin film of (Pr,Ni,Mn)O (PNM5) oxide using an infiltration method. According to the XRD and SEM data, the PNM5 thin film coating, which consisted of a mixture of the Pr_6_O_11_, PrNiO_3_, MnO and NiO oxides, have formed the small particles on the surface of the LSCF backbone particles. The EIS data showed that the PNM5-infiltrated LSCF cathode on the GDC substrate exhibited *R*_p_ = 0.388 Ω cm^2^ at 700 °C compared to *R*_p_ = 1.166 Ω cm^2^ for the bare LSCF cathode. The MPD values of the NiO–DC|GDC|LSCF single cells were equal to 241 and 580 mW cm^−2^ at 750 °C for the cells with the bare and infiltrated LSCF cathodes, respectively. The degradation rates of the above cells were measured to be 0.07093% and 0.02168 at 750 °C during 200 h, respectively. Distribution of Relaxation Times (DRT) function analysis confirmed that the PNM5 coating significantly improved the ORR on the LSCF electrode surface.

Thus, the literature data presented confirm that the infiltration technique is currently the most advanced method for producing active air electrodes with enhanced surface properties.

### 4.4. Improvement of the Electrode–Electrolyte Interface

An advanced air electrode may consist of several layers, each of which has specific requirements. For a functional layer (FL), which is close to the interface with the electrolyte, it is important to have the thermo-mechanical properties (CTE value) close to those of the electrolyte substrate with no chemical interaction with the electrolyte, and high electrochemical activity.

It is known that the electrical conductivity of LSM-based composite layers decreases significantly with increasing electrolyte content [440], so it is necessary to form a collector layer (CL) with high electronic conductivity to ensure uniform current distribution over the electrode volume and to organize a stable current capacity. Therefore, noble metals have been used as the collectors for the composite electrodes: platinum in [149,198,377], and silver in [441]. The oxide electrode collectors with LSM were used in [149,151,387,442] (LSM40), in [205] (LSM30), [442] La_0.65_Sr_0.3_MnO_3 − δ_ (L_0.65_SM30) and in [303,443] (LSM20), [442] ((LS)_0.98_M20).

Harboe et al. [205] investigated the microstructure and electrochemical activity of the L_0.65_SM30–YSZ–graphite–rice starch electrodes with the LSM collector with different compositions in a symmetrical YSZ electrolyte-supported cell. The results obtained showed that the application of the double L_0.65_SM30–YSZ–rice starch electrode combinations with the LSM30 current collector, sintered at 1150 °C, showed the best electrochemical performance of the cathode, equal to 0.26 Ω cm^2^ at 800 °C.

**Figure 10:**
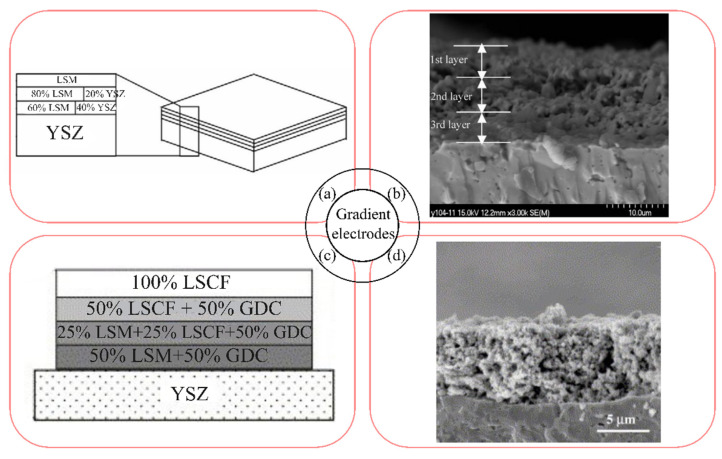
Examples of functional-layer electrodes based on LSM: (**a**) scheme and (**b**) SEM micrograph of three-layer electrode with 60LSM20–40YSZ functional layer, 80LSM20–20YSZ interlayer and LSM20 collector layer (1200 °C, 2 h), obtained by screen printing [379]; (**c**) scheme and (**d**) SEM micrograph of four-layer electrode with 50LSM18–50GDC functional layer, 25LSM18–25LSCF–50GDC and 50LSCF–50GDC interlayers and LSCF collector layer (900 °C, 2 h), obtained by sol–gel slurry deposition ((**a**,**b**) are reproduced from [379] with permission of Springer; (**c**,**d**) are reproduced from [199] with the permission of Elsevier).

In [379] the effect of the configuration of the electrode based on LSM20–YSZ on its performance was investigated. The electrode with an area of 25 mm^2^ was formed by screen printing and annealed at 1200 °C for 2 h. Three electrode configurations were studied: monolayer 80LSM20–20YSZ electrode (the mass ratio of the components in the electrode is shown); bilayer electrode with FL 80LSM20–20YSZ and CL LSM20; triple-layer electrode with FL 60LSM20–40YSZ, interlayer 80LSM20–20YSZ and CL LSM20, as shown in Figure 10a. Scanning electron microscopy showed the absence of any defects at the interfaces between the composite cathode and the electrolyte, and between the different cathode layers for the triple-layer electrode, which was ensured by the gradual change in CTE (Figure 10b). The thickness of each cathode layer was approximately 5–6 μm. The polarization resistances for the above electrode configurations were 0.32, 0.24 and 0.18 Ω cm^2^ at 800 °C, respectively. Maximum values of current density (0.77 A cm^−2^ at an overvoltage of 0.1 V at 800 °C) and specific power of a single SOFC (447 mW cm^−2^ compared to 220 mW cm^−2^ for the single-layer electrode) were obtained for the triple-layer electrode.

Gradient composite electrodes based on La_0.72_Sr_0.18_MnO_3 − δ_ (LSM18), LSCF, GDC were prepared by sol–gel slurry deposition [199]. The polarization resistance values for the 50LSM18–50GDC cathode with the 25LSM18–25LSCF–50GDC and 50LSCF–50GDC interlayers, and the LSCF collector layer (Figure 10c,d), sintered at 900 °C on the YSZ substrate, were equal to 0.21 and 0.10 Ω cm^2^ at 700 and 800 °C, respectively.

Tian et al. [307] fabricated the double-layer electrode LSM35–LSCF with the LSCF top layer infiltrated with SDC, the polarization resistance of which decreased by a factor of 3 times at 800 °C compared to the single-layer LSM electrode. The single fuel cell with the above-fabricated air electrode had an MPD value of 671 mW cm^−2^ at 800 °C, which was 5 times higher than that of the bare LSM cell. The electrolysis cell with the LSM35–LSCF air electrode was characterized by the hydrogen production rate of 547.58 mL cm^−2^ h^−1^ at 800 °C under 1.5 V, which was three times higher than that of the LSM air electrode. The results obtained in [307] justified that the formation of the double-layer air electrodes, whose functional layers consist of both LSCF and LSM, is a promising strategy for enhancing the oxygen reaction reduction and could be useful for the improvement of fuel cells and electrolysis cells.

The addition of La_0.8_Sr_0.2_FeO_3 − δ_ (LSF) into the double functional layer and infiltration were simultaneously used in [444] to fabricate the LSCF−LSF−YSZ composite cathode. Layers of LSF and LSCF were sequentially deposited on the YSZ scaffold with LSF as a protective layer between LSCF and YSZ. The *R*_p_ values of the symmetrical cells with the LSF- and LSCF−LSF-based cathodes were equal to 0.72 and 0.19 Ω cm^2^ at 700 °C, respectively. The MPD values of the single cells with the LSCF−LSF−YSZ and LSF−YSZ cathodes were measured to be 1277 and 922 mW cm^−2^ at 700 °C, respectively. The results obtained showed that the use of the LSF protective layer could be applied to the adoption of the LSCF air electrode for use in YSZ-based cells without CeO_2_-based buffer layers.

The study on the effect of the thickness of FL and CL of the LSCF-based double-layer electrode on its polarization resistance in contact with the GDC10 electrolyte showed that the optimum FL was almost 5 μm-thick, while the optimum CL thickness reached 30 μm [445]. For the symmetrical cell with the optimum thickness cathode layers the excellent *R*_p_ value was measured to be 0.021 Ω cm^2^ at 650 °C.

Lee et al. [186] prepared the LSCF−BCZY15 composite cathode with gradient porosity by a slurry deposition method for use in PCFCs with the BCZY15 electrolyte. The gradient composite cathodes were prepared in such a way that the cathode porosity and cathode powder size increased significantly in the opposite direction from the electrode–electrolyte interface. The polarization resistances of the symmetrical cells with the porosity gradient and the conventional LSCF−BCZY15 composite cathodes were 0.19 and 0.22 Ω cm^2^ at 700 °C, respectively. The MPD values of the NiO–BCZY15|BCZY15|LSCF−BCZY15 single cells with the porosity gradient and the conventional cathodes were 367 and 352 mW cm^−2^ at 700 °C, respectively. In addition, the cell with the porosity gradient cathode showed excellent 100 h stability at 700 °C with a degradation rate of no more than 2%. The authors [186] concluded that it was the gradient structure of the composite cathode that prevented the degradation of the cell performance and stabilized the long-term durability by improving the oxygen adsorption and oxygen reduction at the TPBs.

A numerical simulation of the microstructure of the porosity gradient electrodes was presented in [115]. A “multi-sphere” discrete element method was used to model the powder packing, a kinetic Monte Carlo method was used to model the powder sintering, and the lattice Boltzmann method was used to model the electrochemical reaction at the cathode. It was found that the best performance of the cathode was achieved for the cathode design with a thickness of 25 μm and a density range of 40−65%, which suppressed the sintering but improved the thermal stability of the electrode.

The sintering conditions of the electrode affect the microstructure, adhesion of the electrode to the electrolyte and the possible electrode–electrolyte interdiffusion and chemical interaction, and are, therefore, the most important parameters of the electrode formation.

In [380], the effect of sintering conditions on the performance of electrodes with a 50(LS)_0.95_M25–50YSZ functional layer and an (LS)_0.95_M25 collector was investigated. Electrodes annealed in the temperature range 1150–1300 °C were studied, with the thickness of the FL varying in the range of 4–14 μm, and the collector layer 15–85 μm. Reducing the sintering temperature of the FL resulted in a more porous structure with smaller pores and particles, which reduced the polarization resistance. When the sintering temperature of the FL was kept constant at 1300 °C, a reduction in the annealing temperature of the collector also resulted in a reduction in the polarization resistance. The minimum values of the polarization and contact resistances (0.03 and 0.17 Ω cm^2^ at 1000 °C, respectively) were obtained for the 13 µm thick FL electrode annealed at 1150 °C and the 80 µm thick CL electrode annealed at 1150 °C.

A comparative study to investigate the effect of sintering temperature on the functional properties of (LS)_0.95_M20–YSZ and (LS)_0.95_CF–YSZ composites prepared as hollow fibers in the range of 1250–1450 °C was presented in [241]. For LSCF–YSZ, the formation of the pyrochlore structured impurity phase was observed at the sintering temperature of 1300 °C, followed by the degradation of the perovskite phase at 1400 °C. In contrast, LSM–YSZ showed high thermal stability. It was shown that the highest porosity (34.57% and 33.32% for LSM−YSZ and LSCF−YSZ, respectively) was obtained after sintering at the temperature of 1250 °C, and with the increasing sintering temperature, the porosity and gas permeability of both composites decreased significantly. The mechanical strength of the composites (which is an important property for the cathode-supported SOFCs) was improved by increasing the sintering temperature. For the two composites sintered at 1400 °C and with a sufficient porosity of 22%, the mechanical strength was measured to be 161 and 114 MPa for LSM–YSZ and LSCF–YSZ, respectively. Thus, Ab Rahman et al. [241] concluded that the LSM–YSZ composite cathode was more noticeable at high sintering temperatures compared to the LSCF−YSZ due to its thermal and chemical stability, optimum values of the mechanical strength, gas permeability, and porosity.

The effect of sintering temperature on the performance of the LSCF-based composite cathodes in PCFCs was investigated in [177]. It was shown, that the ohmic and polarization resistances of the LSCF–BZYb composite cathodes on the BZYb electrolyte decreased from 0.76 to 0.45, and from 2.43 to 0.73 Ω cm^2^ at 600 °C for the electrodes sintered at 900 and 1100 °C, respectively. The power densities of the fabricated PCFCs with the LSCF–BZYb composite cathodes sintered at 900 and 1100 °C were equal to 120 and 250 mW cm^−2^ at 600 °C, respectively. Watanabe et al. [177] suggested that a better performance of the electrodes sintered at higher temperatures was due to the improved adhesion at the cathode–electrolyte interface, resulting in an increased number and thickness of the BZYb proton conducting paths within the composite cathodes.

In addition, well-known problems with air electrode delamination [446,447,448] were partially solved by introducing a porous electrolyte layer at the LSM electrode/YSZ dense electrolyte interface [372,384] or by forming nanostructured or contact layers at the LSCF/YSZ interface [449,450]. The flash co-sintering technique was used to form of the LSCF nanofiber coating on the GDC substrate [451] as a possible solution to the electrode delamination problem. The experimental and modeling results showed that severe cracking was observed when the LSCF layer was connected to the electrode. When the LSCF layer was electrically isolated from the electrode, the temperature gradient was dropped. It was concluded that the optimum LSCF/SDC bilayer structure for use in SOFCs would be maintained if the GDC layer density of 92.86% and the LSCF layer porosity of 52.26% were achieved.

He et al. applied the numerical simulation using level-set and adjoint methods to optimize the porous LSCF microstructure [452,453,454]. It was shown that the spherical LSCF solid particles were preferable for performance improvement [452]. In [454], the LSCF/GDC10 electrode–electrolyte interface was numerically modelled using the adjoint method, and it was concluded from the calculation results that the cathode with the optimized electrode–electrolyte interface (optimized electrolyte volume fraction along the cathode thickness direction) had higher electrochemical activity compared to the flat electrode–electrolyte interface.

Thus, the data presented in this section show that the improvement of the perovskite electrode–electrolyte interface aimed at improving the processes occurring in TPBs includes the techniques of the gradient electrode fabrication, the use of collector layers, and the application of the optimum sintering temperature.

## 5. Modeling of the Electrode Performance

The data presented in the above paragraphs justify a greater number of experimental studies to improve the electrochemical performance of the conventional perovskite electrodes. Meanwhile, the high cost of the fuel and electrochemical cell elements and the time required for the investigations have led to the attempts to develop the mathematical models of the electrode performance and to perform 3D reconstruction of solid oxide cells with LSM- and LSCF-based electrodes using computational studies and an Artificial Intelligence (AI) technique. The computational modeling allows the analysis of key parameters that influence the electrochemical activity of the air electrodes.

In [309], Zhang et al. combined the super-resolution of a two-dimensional micrograph with the distribution of relaxation times (DRT) analysis data, showed that the active TPB was the key factor responsible for the LSM–YSZ infiltrated electrode activity, which monitored the electrochemical process at a high frequency. The authors of [309] presented the prospective strategy for modeling three-dimensional heterogeneous nanostructures from two-dimensional graphical data, and proposed an approach for correlating between functional properties of nanostructured electrodes. Sharma et al. [455] simulated a button cell based on a NiO–YSZ fuel electrode, YSZ electrolyte, and LSM air electrode. Electrochemical model equations (Fick’s model, Butler–Volmer equation, Ohm’s law used to estimate the values of concentration, activation, and ohmic overpotential losses, correspondingly) implemented in MATLAB software allowed the overall performance of the cell to be predicted by varying the fuel gas composition, operating temperature, electrolyte thickness and cell configuration. The authors believe that the mathematical model developed in [455] could be a prospect for the future design of solid oxide cells.

**Figure 11:**
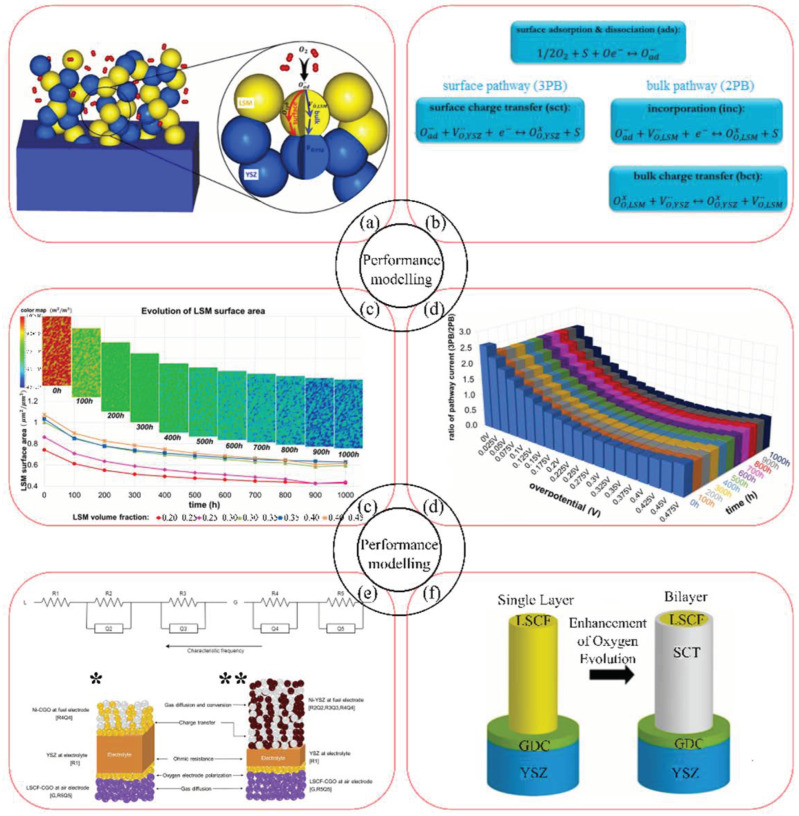
Modeling the degradation of the LSM–YSZ cathode (**a**); 3PB (surface) and 2PB (bulk) pathways of the electrode reaction for LSM–YSZ cathodes (**b**); the predicted evolution trend of the LSM/air interface, where surface adsorption and dissociation steps occur (**c**); the changes in the contributions of the 3PB and 2PB pathways due to structural degradation (**d**); Equivalent Circuit Model for the fit of the impedance data and their attribution to each layer for electrolyte-supported (*) and anode-supported (**) cells with LSCF cathode (**e**); single and bilayer LSCF-based electrode configurations (**f**) ((**a**–**d**) are reproduced from [456]; (**e**) is reproduced from [116], (**f**) is reproduced from [458]).

Yang et al. [456], for the first time, applied a calibrated multiphysics simulation with a multistep ORR model and structural coarsening data from a phase field study to investigate the degradation of the LSM–YSZ composite cathode performance. The multistep oxygen reduction reaction mechanism was considered to involve parallel surface (3PB) and bulk (2PB) pathways (Figure 11a,b). Multiphysical processes, such as charge conservation, gas transport through porous media and surface/bulk transport in the solid phase, were considered. The numerical simulation with a multistep ORR mechanism was simultaneously calibrated with DC polarization curves and AC impedance behavior for different air/fuel supply conditions. The structural changes were found to be sensitive to the volume fraction of LSM in the composite (Figure 11c). The degradation due to grain coarsening in the composite cathode was mainly due to the reduction of active sites for surface and bulk charge transfer steps with a change in the contributions of the 3PB (bulk) and 2PB (surface) pathways (Figure 11d). The simulation results were in good agreement with the experimental investigation of the degradation kinetics of the LSM–YSZ cathode performance carried out using combined isotope exchange, EIS and microstructural study [457].

The numerical simulation of the influence of microstructural characteristics, such as particle size and porosity, on the electrochemical performance of LSCF-based cathodes was provided by the He’s group [459,460]. In [459], it was predicted that the best performance of the LSCF cathode was observed at a porosity of 0.40, and the cathode performance decreased sharply at a critical porosity of 0.10–0.25 due to pore blockage. It was also shown that the LSCF cathodes with the smaller mean pore size could be active at the low porosity of 0.10. In [460], the 3D microstructure of the LSCF–GDC cathode was constructed and the influence of the GDC pillars inside the bare LSCF and composite cathodes with different particle sizes was studied. The results obtained showed that the GDC fibers improved the performance of the LSCF cathode and were more effective in the cathode microstructures with smaller particle sizes.

The prediction of the performance characteristics for the YSZ electrolyte-supported single cell with NiO–SDC as the composite anode and LSCF as the cathode was provided in [461] using a Support Vector Machine (SVM) machine learning technique. The key SOFC parameters, such as the temperature and the supply voltage, were input into the computational model and the values of current density and the MPD values were the output parameters, corresponding to 1160 mA cm^−2^ and 225 mW cm^−2^ at 800 °C, respectively. The prepared NiO–SDC|YSZ|LSCF single cell with hydrogen as fuel showed the values of peak current and power density equal to 1170 mA cm^−2^ and 227 mW cm^−2^ at 800 °C, respectively, which showed the closeness of the theoretically predicted and research data [461].

The experimental and theoretical approaches to identify the contributions of the different cell components were applied in [116] using EIS and a 2D simulation tool for the anode-supported NiO–YSZ|YSZ|LSCF–GDC10 and electrolyte-supported NiO–CGO|YSZ|LSCF–GDC10 planar cells (Figure 11e). It was found that in the electrolyte-supported cell, the largest contribution to the total cell resistance was ohmic due to the greater thickness of the electrolyte layer compared to the anode-supported cell. The major contribution to the total cell resistance of the anode-supported cell was due to the activation overpotential, which was three times higher than that of the electrolyte-supported cell. Gas diffusion was negligible in all tests with the electrolyte-supported cell, in contrast to its large influence in the anode-supported cell. In addition, temperature had a greater influence in the anode-supported cell due to the higher values of the activation energy for the ohmic and activation losses. As the current density increased, the contribution to the activation resistance decreased in both cells studied. Padinjarethil et al. [116] considered that the same work, if improved by introducing the effect of time on the kinetic parameters, could be useful for predicting the long-term operation of SOFCs. The influence of temperature, concentration, and current density gradients within and along the cell structure on the performance of the NiO-cermet anode-supported single cell was evaluated in [462] using a calibration model. The results obtained for the cell in a steady state showed the strong role of the temperature gradients along the channel and the mass transport processes at the gas/electrode interface.

The 1D and 2D models for the LSCF cathode degradation due to sulfur poisoning were developed numerically in [463]. The 1D model viewed a barrier layer formed on the LSCF surface with a random distribution of sulfur content in the thickness direction. The above 1D model was fed to the 2D model of the anode-supported single cell under sulfur poisoning. The results obtained for the 2D simulation model of the planar SOFC showed that it was the model that correctly predicted the SrSO_4_ formation near the cathode–electrolyte interface in the region of the air channel. Iwai et al. concluded that the model proposed in [463] could be used to improve the LSCF cathode by considering the poisoning rate as a function of the barrier thickness.

Modelling of the electrochemical reactions occurring at 2PBs and 3PBs of the single layer La_0.6_Sr_0.4_Co_0.4_Fe_0.6_O_3 − δ_ (LSCF60) and bilayer LSCF60/SCT (strontium cobalt tantalum oxide) air electrodes in a solid oxide electrolysis cell with the YSZ electrolyte and GDC10 buffer layer provided in [458] (as shown in Figure 11f) revealed a competition in electrode kinetics between 2PBs and 3PBs. However, 3PBs were the preferred reactive sites for the single-layer LSCF60 electrode at high voltages. The application of the SCT layer increased the activity of 2PBs, thus reducing the oxygen stoichiometry and dimensional changes. The authors of [458] considered that the analog strategy would be used to prevent delamination at the electrode–electrolyte interface. In addition, the cyclic voltammetry (CV) response of the porous LSCF electrode was simulated in [464], considering the models of solid-state diffusion coupled with oxygen exchange (model-I) and a detailed description of the reaction mechanism (model-II). It was shown that the peaks of the voltammograms were due to the change in oxygen stoichiometry governed by both oxygen diffusion and oxygen exchange, and model-I satisfactorily described the CV curves when the *k*_chem_ constant was determined far from equilibrium. Meanwhile, model II adequately explained the shape of the voltammograms due to the passivation and decomposition of the LSCF surface.

## 6. Conclusions

In the present review, the established and novel methods for modifying conventional air electrodes with perovskite structure were discussed, such as lanthanum strontium manganite La_1 − x_Sr_x_MnO_3 − δ_ (LSM) and lanthanum strontium cobaltite ferrite La_0.6_Sr_0.4_Co_0.8_Fe_0.2_O_3 − δ_ (LSCF), with particular emphasis on the selection of the optimal electrode material composition, electrode layer design, progressive synthesis and deposition methods, and electrode activation techniques. In addition to the creation of defects as the most suitable way to increase the chemical stability of the materials, the development of high-entropy oxides based on LSM and LSCF containing at least five co-dopants was noted. Ceramic methods such as screen printing, tape casting and spraying were found to be the most technologically simple and easily scalable electrode fabrication methods. Spray pyrolysis in its different modifications, along with electrospinning, template method, and 3D printing were mentioned among the variety of methods available to improve the conventional perovskite electrodes by extending TPB. The deposition of protective and non-catalytic layers on the electrode surface was proposed as a solution to the problems of segregation, impurity poisoning and sluggish oxygen kinetics.

New trends in the design of the LSM- and LSCF-based composite electrodes were highlighted, including various infiltration techniques, modification of the electrode structure using nanofibers and the creation of core–shell structures. The selection of the optimum sintering temperature, the creation of the electrodes with gradient composition and porosity and the use of collector layers were shown to be important in improving the perovskite electrode–electrolyte interface, preventing electrode delamination, and ensuring stable current collection.

It was shown that computational design and numerical simulations of the perovskite-based composite allowed us to suggest possible degradation mechanisms and to understand the electrode behavior at both atomic and macroscopic scales. These methods were found to be useful for quickly analyzing key parameters that influence the electrochemical activity of the air electrodes and for predicting the durability of the electrode without time-consuming experimental work. The development of the mathematical models was shown to be promising for the future design of solid oxide cells.

The present review, which summarizes the basic information on the methods to improve the electrochemical performance of conventional air electrodes, can serve as a guide for the application of the developed technologies to the modification of electrodes with novel, recently investigated electrode materials.

## Data Availability

Not applicable.
