# Correction: Filonova, E.; Pikalova, E. Overview of Approaches to Increase the Electrochemical Activity of Conventional Perovskite Air Electrodes. *Materials* 2023, *16*, 4967

**DOI:** 10.3390/ma16186278

**Published:** 2023-09-19

**Authors:** Elena Filonova, Elena Pikalova

**Affiliations:** 1 Department of Physical and Inorganic Chemistry, Institute of Natural Sciences and Mathematics, Ural Federal University, Yekaterinburg 620002, Russia; 2 Laboratory of Kinetics, Institute of High Temperature Electrochemistry, Ural Branch of the Russian Academy of Sciences, Yekaterinburg 620137, Russia; e.pikalova@list.ru; 3 Department of Environmental Economics, Graduate School of Economics and Management, Ural Federal University, Yekaterinburg 620002, Russia

## Missing Citations

In the original publication [1], four references were not cited. These citations as references [225–228] have now been inserted into the caption in Figure 5 and should read as follows:

**Figure 5.** Schemes of methods used to apply electrode layers: (**a**) slurry application; (**b**) spraying; (**c**) screen printing; (**d**) film casting ((**a**) is reproduced from [225] with permission of IOP Publishing; (**b**) is reproduced from [226] with permission of Taylor and Francis; (**c**) is reproduced from [227]; (**d**) is reproduced from [228] with permission of J. Wiley and Sons).

225. Ahamad, S.; Ahmad, M.; Mehta, B.R.; Gupta, A. Effect of Nano-Fillers on Capacity Retention and Rate Capability of Mesocarbon Microbeads Anode. *J. Electrochem. Soc.* **2017**, *164*, A2967–A2976. https://doi.org/10.1149/2.0351713jes.

226. Verma, R.; Suri, N.M.; Kant, S. Effect of Parameters on Adhesion Strength for Slurry Spray Coating Technique. *Mater. Manufact. Proc.* **2017**, *32*, 416–424. https://doi.org/10.1080/10426914.2016.1221090.

227. Creanga, C.; Serban, S.; Pittson, R.; Murr, N.E. “No Calibration” Type Sensor in Routine Amperometric Bio-sensing: An Example of a Disposable Hydrogen Peroxide Biosensor. In *Biosensors—Emerging Materials and Applications*; InTechOpen: London, UK, 2011; pp. 141–152. https://doi.org/10.5772/17769.

228. Ren, L.; Luo, X.; Zhou, H. The Tape Casting Process for Manufacturing Low-temperature Co-fired Ceramic Green Sheets: A Review. *J. Am. Ceram. Soc.* **2018**, *101*, 3874–3889. https://doi.org/10.1111/jace.15694.

References [225–460] in the original publication would be updated to [229–464] accordingly.

The authors state that the scientific conclusions are unaffected. This correction was approved by the Academic Editor. The original publication has also been updated.

## References

[1] Filonova E., Pikalova E. (2023). Overview of Approaches to Increase the Electrochemical Activity of Conventional Perovskite Air Electrodes. Materials.

